# Role of hippo pathway and cuproptosis-related genes in immune infiltration and prognosis of skin cutaneous melanoma

**DOI:** 10.3389/fphar.2024.1344755

**Published:** 2024-03-07

**Authors:** Haozhen Lv, Lin Liu, Yuexi He, Kun Yang, Yu Fu, Yingqiu Bao

**Affiliations:** ^1^ Department of Dermatology, Beijing Hospital, National Center of Gerontology, Institute of Geriatric Medicine, Chinese Academy of Medical Sciences, Beijing, China; ^2^ Peking Union Medical College, Beijing, China

**Keywords:** cuproptosis, melanoma, Yap1, prognosis, immune infiltration

## Abstract

Melanoma is the most lethal type of skin cancer with an increasing incidence. Cuproptosis is the most recently identified copper-dependent form of cell death that relies on mitochondrial respiration. The hippocampal (Hippo) pathway functions as a tumor suppressor by regulating Yes-associated protein/transcriptional coactivator with PDZ-binding motif (YAP/TAZ) activity. However, its role in cuproptosis remains unknown. In addition, the correlation of cuproptosis-related genes and Hippo pathway-related genes with tumor prognosis warrants further investigation. In the present study, we explored the correlation of cuproptosis-related genes and Hippo pathway-related genes with the prognosis of melanoma through analysis of data from a public database and experimental verification. We found eight Hippo pathway-related genes that were downregulated in melanoma and exhibited predictive value for prognosis. There was a significant positive correlation between cuproptosis-related genes and Hippo pathway-related genes in skin cutaneous melanoma. YAP1 expression was positively correlated with ferredoxin 1 (FDX1) expression in the GSE68599 dataset and A2058 cells. Moreover, YAP1 was positively and negatively correlated with M2 macrophages and regulatory T cell infiltration, respectively. In conclusion, the present study demonstrated the prognostic value of Hippo pathway-related genes (particularly YAP1) in melanoma, revealing the correlation between the expression of Hippo pathway-related genes and immune infiltration. Thus, the present findings may provide new clues on the prognostic assessment of patients with melanoma and a new target for the immunotherapy of this disease.

## 1 Introduction

Skin cutaneous melanoma (SKCM) is caused by the malignant transformation of melanocytes. It is the most aggressive form of skin cancer, associated with high mortality and rapid metastatic potential (Arnold et al., 2022). Early melanoma can be treated with surgery; however, advanced melanoma has been linked to a poor prognosis and cannot be managed through surgery. Recently, immune checkpoint blockade has shown a great prospect in treating a wide variety of cancers, including lymphoma, melanoma, and squamous non-small cell lung cancer (Alencar and Moskowitz, 2019). The treatment of melanoma has improved dramatically over the past decade. Unfortunately, due to the resistance to current therapies and recurrence of melanoma, the prognosis of melanoma remains unoptimistic (Ding et al., 2019). Therefore, it is imperative to uncover the molecular mechanisms responsible for the pathogenesis of cutaneous melanoma. This knowledge would assist in the development of preventive measures and curative treatment options.

Cuproptosis is a recently discovered form of cell death; it is distinct from other known types of cell death, such as apoptosis, ferroptosis, and necroptosis (Tsvetkov et al., 2022). Copper is a type of trace metal essential for life. Proper regulation of copper levels plays a crucial role in maintaining the function of all organisms and homeostasis. Increased copper levels have been detected in various malignancies, stimulating the proliferation, angiogenesis, and metastasis of tumor cells (Oliveri, 2022). Copper deficiency impairs the function of copper-binding enzymes, whereas copper accumulation induces cellular death (Kahlson and Dixon, 2022). As reported by Tsvetkov et al., cuproptosis dependent on mitochondrial respiration regulated by targeting lipoylated components of the tricarboxylic acid (TCA) cycle. They found seven genes (FDX1, LIAS, LIPT1, DLD, DLAT, PDHA1 and PDHB) conferred resistance to cuproptosis, while three genes (MTF1, GLS, and CDKN2A) sensitized the cells to cuproptosis through whole-genome CRISPR-Cas9 selection screen (Tsvetkov et al., 2022). The Fe–S cluster protein FDX1 is key regulator of cuproptosis among these cuproptosis related genes (Huo et al., 2023). FDX1 was considered act as the upstream of LA pathway, and under the regulation of FDX1, LIAS linked lipoyl moiety to DLAT, which was essential for the function of mitochondria. Recent study found a significant difference in the expression of copper death-related genes between SKCM and normal tissues, suggesting an association between cuproptosis and the occurrence of SKCM. Additionally, they indicate that prognostic model constructed based on cuproptosis-related genes can effectively predict prognosis information for SKCM patients, highlighting the value of copper death-related genes in melanoma prognosis prediction (Qin et al., 2023). Zhou et al. also develop a novel risk model based on cuproptosis-related lncRNAs, which predicted prognosis and indicated immune microenvironment landscape of patients with SKCM (Zhou et al., 2022).

The hippocampal (Hippo) pathway functions as a tumor suppressor by regulating Yes-associated protein/transcriptional coactivator with PDZ-binding motif (YAP/TAZ) activity. Dysregulation of this pathway has been implicated in the development and progression of various cancer types, including cutaneous malignancies (Rognoni and Walko, 2019). It is a crucial regulator of organ size discovered through studies on *Drosophila* tissue growth (Tapon et al., 2002). This pathway is not necessary for normal tissue homeostasis; nevertheless, it is essential in melanoma (Andl et al., 2017). It has been shown that inactivation of the Hippo pathway promotes the growth and invasion of cutaneous melanoma (Zhang et al., 2019; Zhang et al., 2020). The Hippo pathway enforces stable growth arrest of nevus melanocytes, which represents a significant barrier to the development of melanoma. Dysregulation of the Hippo pathway enables oncogenic BRAF-expressing melanocytes to bypass nevus formation and rapidly progress into malignant melanomas (Vittoria et al., 2022). Thus, the Hippo pathway represents a promising therapeutic target in a subset of melanomas; however, the role of Hippo signaling in cuproptosis of SKCM is poorly defined.

YAP1 is recognized as the downstream oncogene of the Hippo pathway (Wu et al., 2018). Proteins involved in the Hippo pathway modulate the nuclear localization of transcription co-activators YAP and TAZ to regulate organ size. Large tumor suppressor kinase 1/2 (LATS1/2) phosphorylate serine residues (at least five in YAP) to repress YAP and TAZ, leading to their binding with 14-three to three proteins and cytoplasmic sequestration. YAP and TAZ, particularly proteins belonging to the TEA domain transcription factors (TEAD) family, modulate gene expression via transcription factors. While YAP1 is not frequently mutated in human melanoma, amplifications and mutations of YAP1 have been observed. Menzel et al. found that copy number gains directly affected YAP in 4%–10% of patients, while the copy number of known Hippo pathway-related genes was affected in 62% of patients with melanoma (Menzel et al., 2014). A significant correlation between YAP staining and reduced patient survival in primary melanoma has been discovered (Menzel et al., 2014; Shain et al., 2015; Zhang et al., 2019).

Cuproptosis, as a newly discovered form of cell death, holds significant research value in SKCM. Meanwhile, the Hippo pathway, known for its importance in various cancers, including SKCM, has unclear mutual regulation with the occurrence of cuproptosis. Therefore, in this study, we conducted a systematic investigation of the association between cuproptosis-related genes and the Hippo pathway in patients with SKCM, based on an analysis of data obtained from The Cancer Genome Atlas (TCGA) database and Gene Expression Omnibus (GEO) database. We propose that the expression of Hippo pathway genes is aberrant and positively correlated with the expression of copper death genes in SKCM. Combining the two pathway related genes, a prognostic model constructed demonstrates an effective differentiation of prognosis among SKCM patients. Regulating YAP1 can influence the expression of key genes in cuproptosis, suggesting their potential as therapeutic targets for SKCM.

## 2 Materials and methods

### 2.1 Data acquisition

**FIGURE 1:**
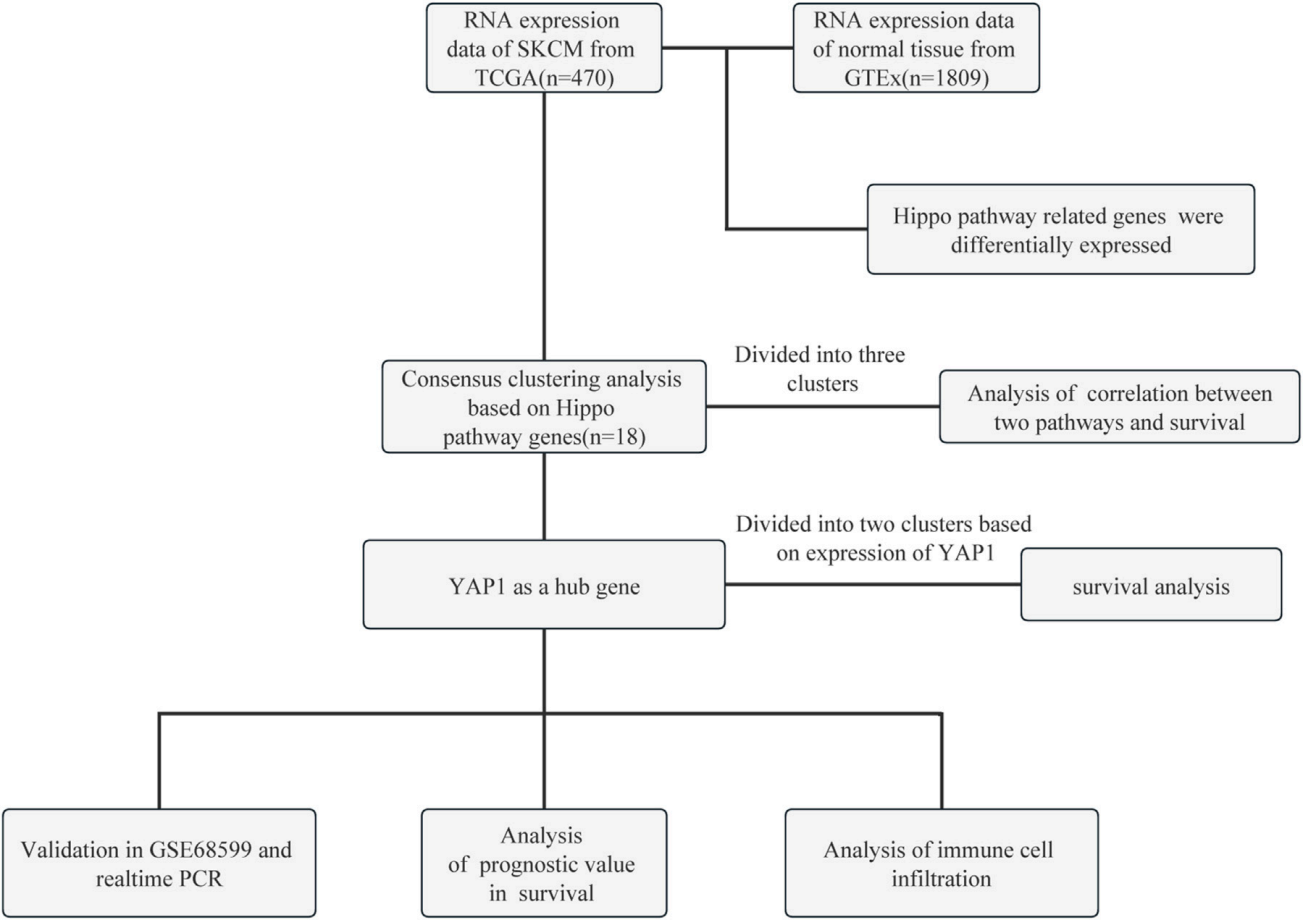
Flow chart of this study. GTEx, Genotype-Tissue Expression; PCR, polymerase chain reaction; SKCM, skin cutaneous melanoma; TCGA, The Cancer Genome Atlas; YAP1, Yes-associated protein 1.

The flow chart of this study is shown in Figure 1. The RNA-sequencing expression profiles of 470 patients with SKCM were acquired from the TCGA dataset available at the portal of the Genomic Data Commons (https://portal.gdc.com). Normal tissue samples were obtained from the Genotype-Tissue Expression (GTEx) data portal (https://www.gtexportal.org/home/datasets). Additionally, the GSE68599 dataset from the GEO (https://www.ncbi.nlm.nih.gov/geo/) database was downloaded and utilized for further validation of the impact of YAP1 regulation on the expression levels of cuproptosis-related genes.

### 2.2 Subgroup analysis

To assess consistency, the “ConsensusClusterPlus” R package (version 1.54.0) was employed for subgroup analysis. A maximum of six clusters could be formed, and 80% of the total sample was drawn 100 times using the hierarchical clustering algorithm with the inner linkage method of wardD2. Genes with a standard deviation >0.1 were retained based on the gene expression heatmap. The top 25% of genes, sorted by standard deviation, were extracted. Samples from the TCGA database were categorized into three clusters based on the expression levels of Hippo pathway-related genes in patients with SKCM.

### 2.3 Immune score analysis

To evaluate the reliability of immune score assessment, we utilized the “CIBERSORT” algorithm, which is a recent addition to the “immunedeconv” package. The immune score analysis in this study utilized various analytical methods and R packages, including R (version 4.0.3) and software packages “ggplot2” and “pheatmap”.

### 2.4 Survival analysis

Log-rank tests and univariate Cox proportional hazards regression were employed to generate Kaplan–Meier curves, *p*-values, and hazard ratios with a 95% confidence intervals. The hazard ratio serves as an indicator of the role of a gene as a risk factor (expression <1.0) or a protective factor (expression >1.0). Additionally, R packages “ggsrisk”, “survival”, “survminer”, and “timeROC” were utilized (version 4.0.3). The *p*-values <0.05 indicate statistically significant differences.

### 2.5 Cell culture and quantitative real-time polymerase chain reaction (qRT-PCR)

This study was approved by the Ethics Committee of Beijing Hospital (approval number: 2023BJYYEC-152-01). Primary melanocytes (PMC) were obtained from the foreskin and cultured in MelM-2 (No. 2211, ScienCell). The culture medium was supplemented with melanocyte growth supplement and 2.5 mL of fetal bovine serum. A2058 cells were cultured in Dulbecco’s modified Eagle’s medium supplemented with 10% fetal calf serum and 100 units/mL of penicillin (both obtained from Thermo Fisher, United States). Melanocytes in the second and third passages were transferred into a six-well plate. RNA extraction was performed using an RNA extraction kit (ES Science, Shanghai, China). The RNA was quantified and subjected to reverse transcription to generate complementary DNA. The qRT-PCR was conducted using SYBR Green fluorescence (TaKaRa, Japan). Fold changes in expression were determined relative to the levels of reference gene PMC using the delta-delta Ct method.

### 2.6 Construction of the prognosis signature in melanoma

RNA-sequencing expression profiles and corresponding clinical data for SKCM were obtained from the TCGA dataset (https://portal.gdc.com). The count data were converted to transcripts per million (TPM) and normalized using the log2 (TPM+1) transformation. Only samples with available clinical information were retained, resulting in a final sample size of 456 for subsequent analysis. The log-rank test was employed to assess disparities in survival rates among the aforementioned groups. The timeROC (version 0.4) analysis was utilized to compare the prognostic accuracy of genes and risk scores. Feature selection was performed using the least absolute shrinkage and selection operator (LASSO) regression algorithm, with 10-fold cross-validation and analysis conducted using the R package glmnet.

### 2.7 Statistical analysis

The statistical distinction between two groups was evaluated using the Wilcoxon test. Kaplan–Meier survival analysis was conducted based on the gene signature obtained from the TCGA dataset. Comparisons among different groups were carried out using the log-rank test. The predictive value of genes for overall survival (OS) was assessed through the utilization of receiver operating characteristic (ROC) curves, employing the “timeROC” package. Univariate and multivariate Cox regression analyses were conducted to determine the appropriate variables for constructing the nomogram. Statistical analyses were carried out using R software. A *p*-value <0.05 indicates a statistically significant difference.

## 3 Results

### 3.1 Hippo pathway-related genes were differentially expressed between normal biopsies and SKCM

**FIGURE 2:**
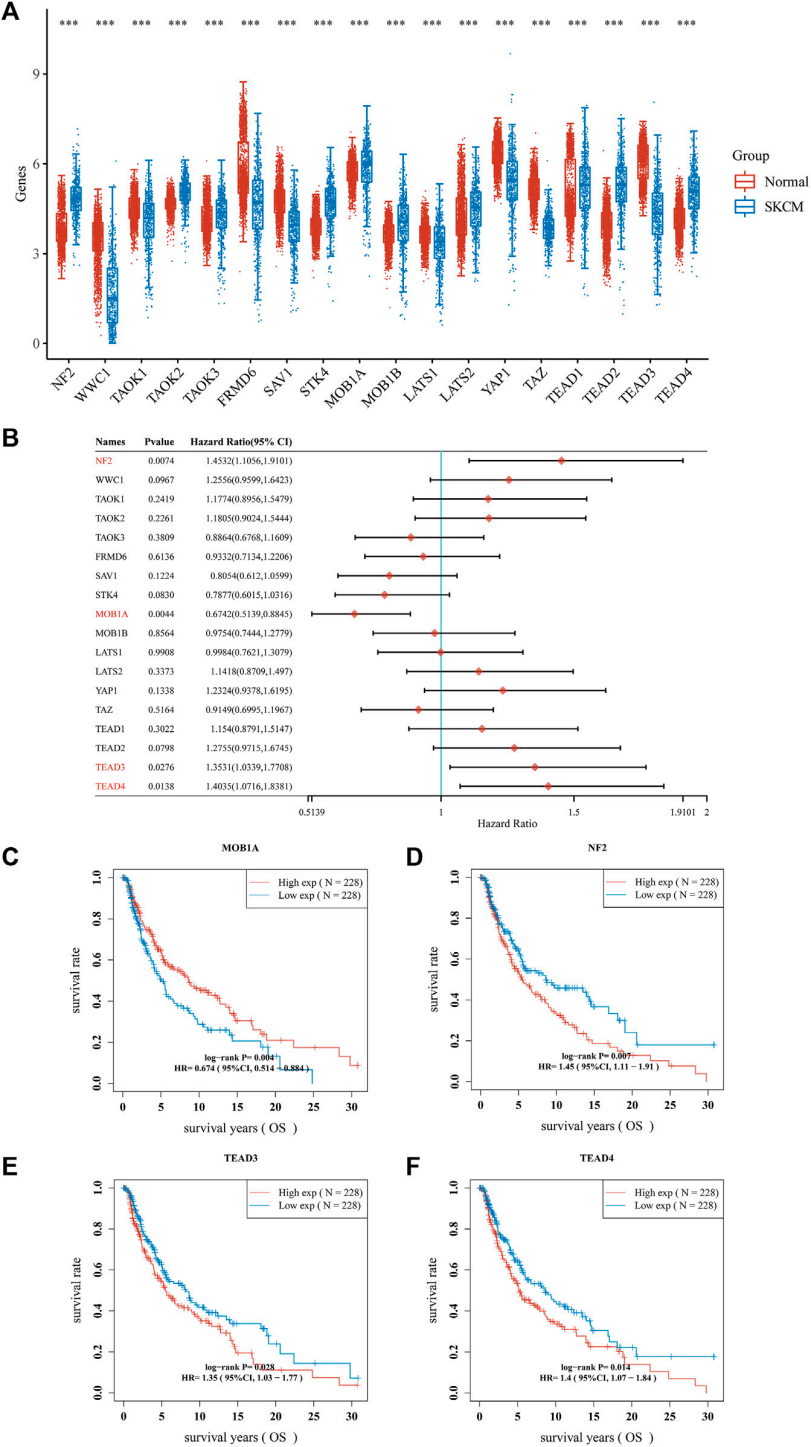
Hippo pathway-related genes expressed in patients with SKCM and their prognostic value. **(A)** Box plots of Hippo pathway-related genes in SKCM compared with normal tissues. **(B–F)**. Nomograms and Kaplan–Meier survival analysis of Hippo pathway-related genes in SKCM established using univariate analyses. ***p < 0.001. CI, confidence interval; HR, hazard ratio; OS, overall survival; SKCM, skin cutaneous melanoma.

Dysregulation of the Hippo pathway is implicated in numerous types of cancer, regulating various key aspects of the disease. It has been demonstrated that 18 genes (NF2, WW and C2 domain containing 1 [WWC1], TAO kinase 1 [TAOK1], TAOK2, TAOK3, FERM domain containing 6 [FRMD6], salvador family WW domain containing protein 1 [SAV1], serine/threonine kinase 4 [STK4], MOB kinase activator 1A [MOB1A], MOB1B, LATS1, LATS2, YAP1, TAZ, TEAD1, TEAD2, TEAD3, and TEAD4) are associated with the Hippo pathway. We sought to verify the involvement of these Hippo pathway-related genes in melanoma. Therefore, we conducted a comparative analysis of their expression patterns between melanoma and adjacent normal tissues obtained from TCGA and GTEx databases. We analyzed 470 melanoma tissues and 1,809 adjacent normal tissues. The aforementioned 18 genes exhibited differential expression between melanoma and adjacent normal tissues (Figure 2A). Among these differentially expressed genes, 10 genes (NF2, TAOK2, TAOK3, STK4, MOB1A, MOB1B, LATS2, TEAD1, TEAD2 and TEAD4) were upregulated, whereas eight genes (WWC1, TAOK1, FRMD6, SAV1, LATS1, YAP1, TAZ and TEAD3) were downregulated in melanoma (Figure 2A).

The prognostic significance of Hippo pathway-related genes in melanoma was further assessed. We conducted Cox regression analysis on the 18 hippo pathway-related genes and presented the results in the form of a forest plot (Figure 2B). Among the 18 genes, the expression of four genes (MOB1A, NF2, TEAD3 and TEAD4) showed a significant correlation with prognosis. As shown in Figure 2C, patients with melanoma expressing higher MOB1A levels were linked to longer OS than those expressing lower MOB1A levels (Figure 2C). In addition, patients with melanoma who expressed lower levels of NF2, TEAD3, and TEAD4 were associated with longer OS than those who expressed lower levels of NF2, TEAD3, and TEAD4 (Figures 2D–F).

### 3.2 Consensus clustering analysis of hippo pathway-related genes identified differences in baseline characteristics and survival between three melanoma subgroups

**FIGURE 3:**
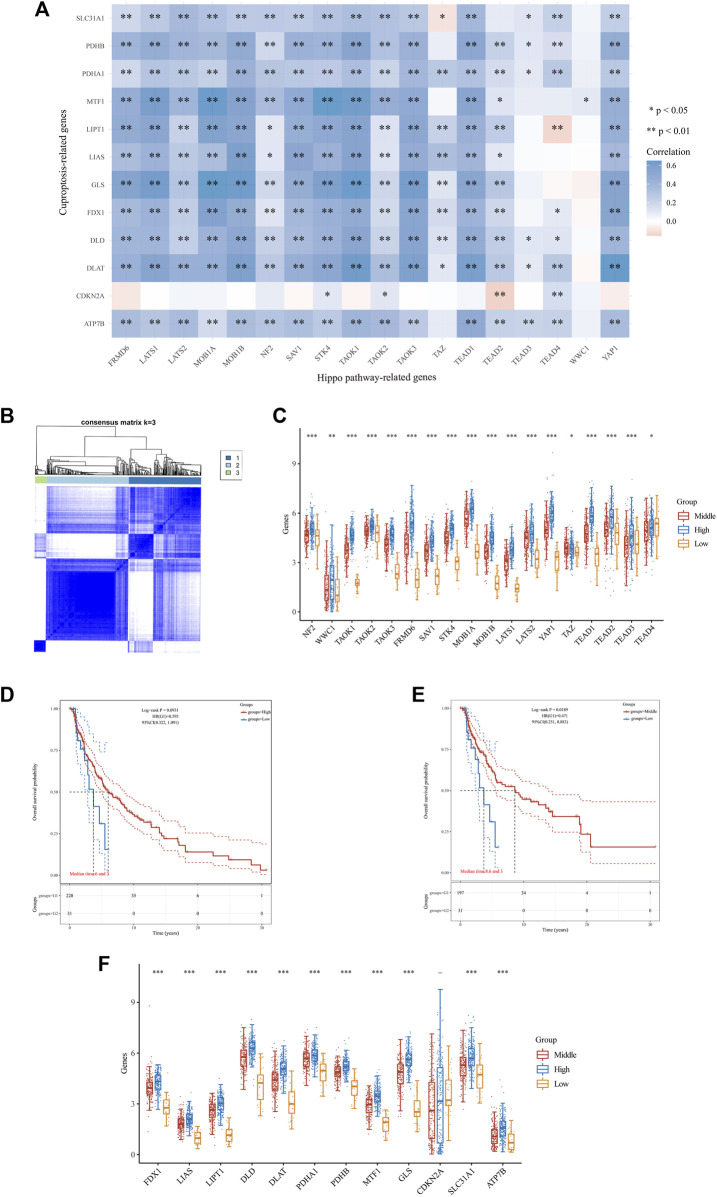
Differential expression pattern of Hippo pathway-related genes, survival, and functional enrichment analysis in three skin cutaneous melanoma (SKCM) clusters. **(A)** Correlation between Hippo pathway-related genes and genes related to copper-induced cell death in SKCM. **(B)** Consensus clustering matrix for k = 3. **(C)** Box plots visualizing the expression patterns of Hippo pathway-related genes in three SKCM clusters. **(D,E)** Kaplan–Meier curves showing overall survival for three clusters of patients with SKCM. **p < 0.01, and ***p < 0.001. **(F)** Box plots visualizing the expression patterns of genes related to copper-induced cell death in three SKCM clusters.

Previous studies have indicated the involvement of 12 genes (FDX1, LIAS, LIPT1, DLD, DLAT, PDHA1, PDHB, MTF1, GLS, CDKN2A, SLC31A1 and ATP7B) in regulating copper-induced cell death, and their abnormal expression in SKCM (Tsvetkov et al., 2022; Qin et al., 2023). Our research also identified abnormal expression of 18 Hippo pathway-related genes in SKCM (Figure 2A). Whether there is a correlation between Hippo pathway-related genes and c uproptosis-related genes remains unknown. Therefore, we first investigated the correlation between the expression levels of them in SKCM through spearman’s correlation analysis. The correlation and prognosis analyses revealed a positive association between the expression of c uproptosis-related genes and Hippo pathway-related genes (Figure 3A).

The consensus clustering analysis was conducted using the Consensus Cluster Plus package in R software. The optimal clustering stability was selected to be k = 3 in consensus clustering (Figure 3B). As a result, the cohort of 470 patients with melanoma obtained from TCGA database was stratified into three distinct subgroups. The expression levels of 16 Hippo pathway-related genes (NF2, WWC1, TAOK1, TAOK2, TAOK3, FRMD6, SAV1, STK4, MOB1A, MOB1B, LATS1, LATS2, YAP1, TEAD1, TEAD2, and TEAD3) were higher in the high cluster *versus* the middle and low clusters (Figure 3C). Additionally, patients in the high and middle clusters had markedly longer OS than those in the low cluster (Figures 3D, E). Moreover, we found that higher expression of Hippo-related genes was associated with higher levels of copper-induced cell death-related genes (Figure 3F). These findings indicated that Hippo pathway-related genes may inhibit the progression of melanoma by regulating molecular processes and pathways related to copper-induced cell death.

### 3.3 Confirmation of YAP1 expression in patients with melanoma and bioinformatics analysis of YAP1-related gene signatures

**FIGURE 4:**
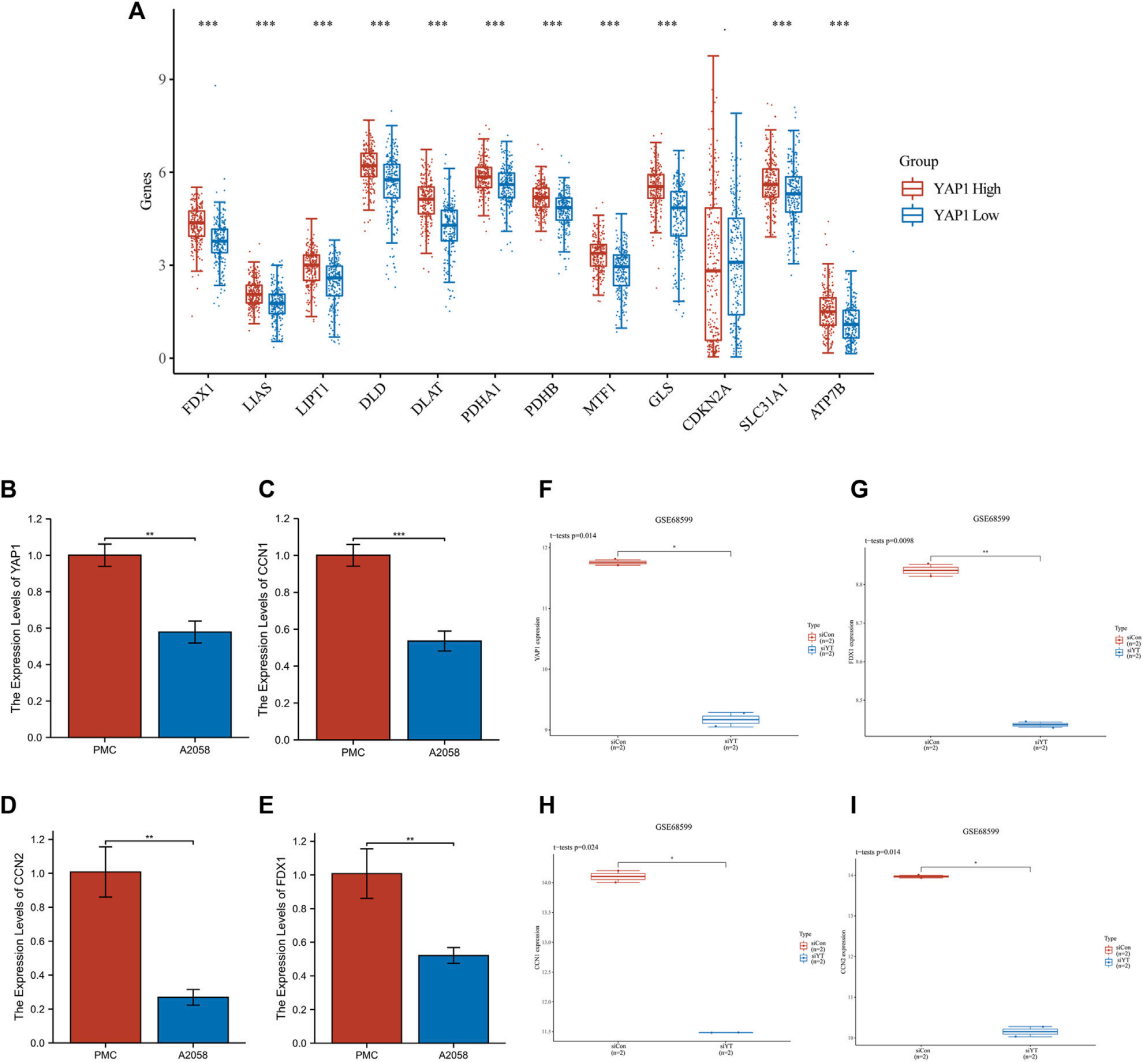
Analysis of YAP1 gene expression in SKCM. **(A)** The expression of genes related to copper-induced cell death was higher in the YAP1-high cluster *versus* the YAP1-low cluster in SKCM. **(B–D)** Changes in YAP1, CCN1, CCN2, FDX1 gene expression assessed by real-time qPCR. **(F–I)** Transfection of resistant WM3248 cells with siYAP1 downregulated the expression of CCN1, CCN2, FDX1 in GSE68599. CCN1/2, cellular communication network factor 1/2; FDX1, ferredoxin 1; SKCM, skin cutaneous melanoma; YAP1, Yes-associated protein 1.

The aforementioned 18 genes were associated with the Hippo pathway and differentially expressed between normal biopsy tissues and SKCM. Among them, YAP1 is the pivotal effector of the Hippo signaling pathway, playing a vital role in Hippo signaling (Zhang et al., 2019; Chen et al., 2020). In the present study, analysis of data downloaded from TCGA database revealed that YAP1 was positively associated with genes related to copper-induced cell death in patients with melanoma. Therefore, we further investigated the role of YAP1 in the development of melanoma. For this purpose, we stratified patients into two subgroups based on their YAP1 expression levels, namely, high- and low-YAP1 groups. Eleven genes related to copper-induced cell death were upregulated in the high-YAP1 group compared with the low-YAP1 group (Figure 4A). The qRT-PCR results confirmed that the expression levels of YAP1, cellular communication network factor 1 (CCN1), and CCN2 were significantly downregulated in A2058 cells compared with PMC cells (Figures 4B–D).

Since FDX1 is crucial for cuproptosis, we questioned whether YAP1 regulates the expression of cuproptosis-related genes. Thus, we downloaded data (GSE68599), and analyzed the expression of FDX1, YAP1, CCN1, and CCN2 in resistant WM3248 cells. The siRNA approach was used to inhibit the expression of YAP1 in resistant WM3248 cells. Following the downregulation of YAP1 through transfection of resistant WM3248 cells with siYAP1, the expression levels of FDX1, CCN1, and CCN2 were significantly decreased compared with those measured in control cells (Figures 4F–I). This evidence showed that YAP1 can regulate cuproptosis in SKCM. The qRT-PCR results also confirmed that the expression levels of FDX1 were significantly downregulated in A2058 cells compared with PMC cells (Figure 4E). Furthermore, we also analyzed the expression of other cuproptosis-related genes after inhibiting YAP1. It was observed that the expression of LIAS, DLAT, DLD, and PDHA1 decreased with the inhibition of YAP1; however, the differences were not significant (*p* > 0.05) (Supplementary Figures S2A-D).

### 3.4 Evaluation of the prognostic value of the hippo pathway and cuproptosis in patients with melanoma

The prognostic value of upregulated Hippo pathway-related genes and cuproptosis-related genes in melanoma was further evaluated. Two prognostic signatures for melanoma were constructed using Hippo pathway-related genes and cuproptosis-related genes. The coefficients of selected features are shown by the lambda parameter. The abscissa represents the value of lambda, and the ordinate represents the coefficients of the independent variable (Supplementary Figures S1A, B). First of all, ten prognosis-related key Hippo pathway-related genes were identified and integrated to construct a prognostic signature (model1) for melanoma.

The risk score was calculated as follows:

$$\begin{array}{c} \text{Risk score}=\left(0.0341*\text{NF}2\right)+\left(0.0769*\text{WWC}1\right)+\left(‐0.037*\text{SAV}1\right)+\left(‐0.0443*\text{STK}4\right)+\left(‐0.1713*\text{MOB}1A\right)\\ \;+\left(0.0419*\text{LATS}2\right)+\left(0.0062*\text{YAP}1\right)+\left(‐0.0887*\text{TAZ}\right)+\left(0.1326*\text{TEAD}3\right)+\left(0.1854*\text{TEAD}4\right) \end{array}$$

**FIGURE 5:**
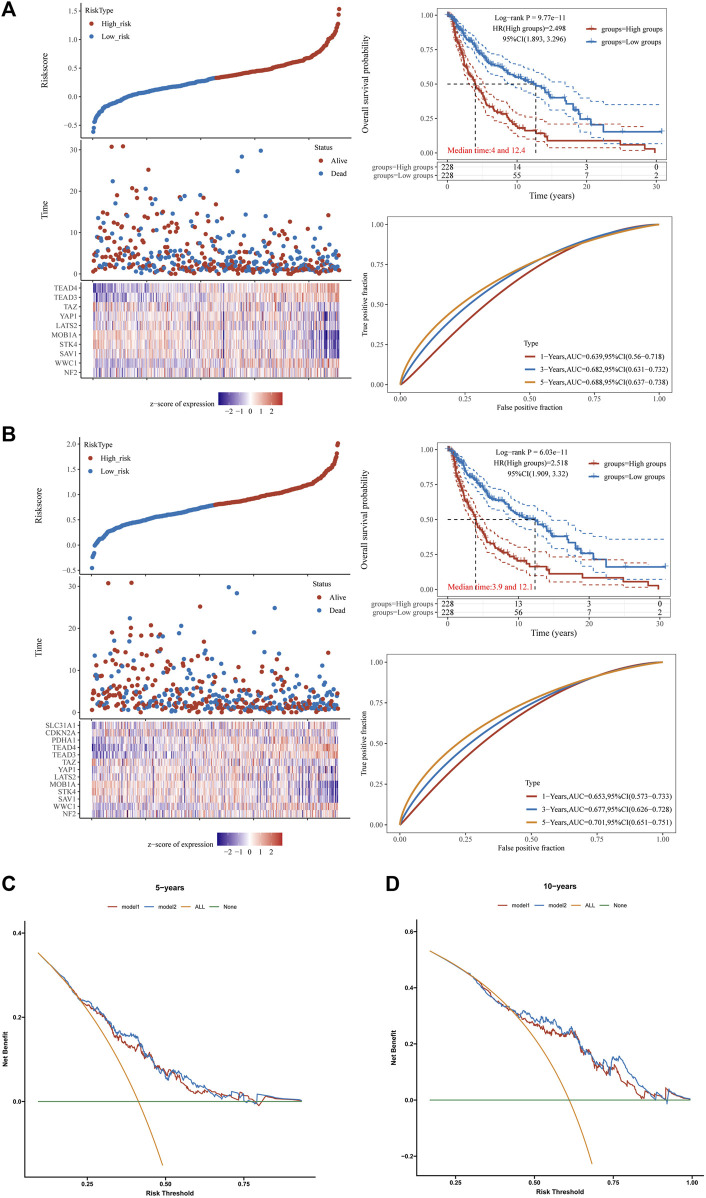
Evaluation of the prognostic value of the Hippo pathway and cuproptosis in skin cutaneous melanoma (SKCM). The risk score, survival time, and survival status based on a signature composed of Hippo pathway-related genes **(A)**, and prognostic value of Hippo pathway- and cuproptosis-related genes **(B)** in SKCM. The top scatterplot represents the risk score from low to high values. Different colors represent different groups. The scatter plot distribution represents the risk score of different samples corresponding to the survival time and survival status. The bottom heatmap shows gene expression according to the signature. **(C,D)** Decision curve analysis of candidate mRNAs for predicting survival status of two signatures.

Patients were stratified into high- and low-Hippo pathway risk score groups according to the selected cut-off value. As shown in Figure 5A, patients in the high-risk group were more likely to survive than those in the low-risk group. Additionally, patients in the high-risk group had longer OS than those in the low-risk group (Figure 5A). Subsequently, the efficiency of Hippo pathway-related genes for predicting the prognosis of patients with melanoma receiving immunotherapy was evaluated through ROC curve analysis. The area under the curve values for predicting 1-, 3-, and 5-year survival were 0.639, 0.682, and 0.688, respectively (Figure 5A).

The previous analysis showed that Hippo pathway-related genes were positively correlated with cuproptosis-related genes in melanoma. This suggests prognostic model1 constructed based on the Hippo pathway may not be perfect. Simultaneously, integrating a prognostic model constructed from both Hippo pathway and cuproptosis-related genes may enhance the predictive ability for prognosis. Next, we added cuproptosis-related genes (pyruvate dehydrogenase E1 subunit alpha 1 [PDHA1], cyclin dependent kinase inhibitor 2A [CDKN2A], solute carrier family 31 member 1 [SLC31A1]) to the prognostic signatures to construct a more effective prognostic signature (model2) for melanoma.

The risk score was calculated as follows:

$$\begin{array}{c} \text{Risk score}=\left(0.0216*\text{NF}2\right)+\left(0.0846*\text{WWC}1\right)+\left(‐0.0933*\text{SAV}1\right)+\left(‐0.0461*\text{STK}4\right)+\left(‐0.1995*\text{MOB}1A\right)\\ \;+\left(0.0681*\text{LATS}2\right)+\left(0.0098*\text{YAP}1\right)+\left(‐0.1645*\text{TAZ}\right)+\left(0.1407*\text{TEAD}3\right)+\left(0.1706*\text{TEAD}4\right)\\ \;+\left(0.1759*\text{PDHA}1\right)+\left(‐0.0223*\text{CDKN}2A\right)+\left(0.0275*\text{SLC}31A1\right) \end{array}$$

The area under the curve values for predicting 1-, 3-, and 5-year survival were 0.653, 0.677, and 0.701, respectively (Figure 5B). Then we employed the decision curve analysis (DCA analysis) method to assess and compare the differences between the two models. The newly constructed prognostic model (model2) integrating both the Hippo pathway and cuproptosis-related genes demonstrates superior capability in predicting 5-year and 10-year survival periods for SKCM patients compared to the prognostic model based solely on the Hippo pathway-related genes (model1) (Figures 5C, D). This suggests that model2 has better clinical value in predicting patient survival compared to model1.

### 3.5 Correlation analysis of YAP1 expression with immune cell infiltration

**FIGURE 6:**
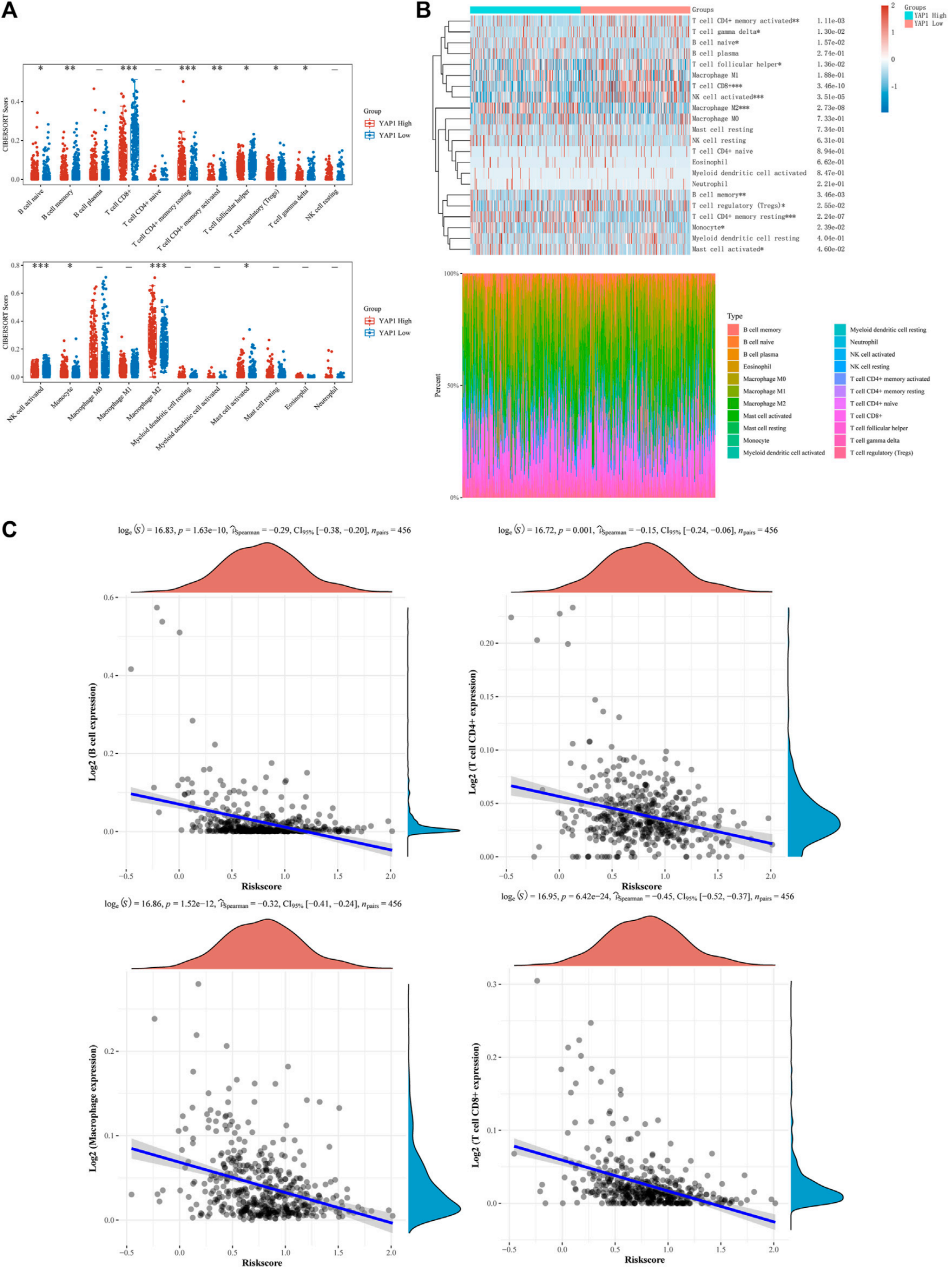
Relationship between YAP1 expression and immune cell infiltration in skin cutaneous melanoma (SKCM) by correlation analysis. **(A)** Levels of immune cell infiltration in high- and low-YAP1 expression groups of patients with SKCM. **(B)** The percentage abundance of tumor-infiltrating immune cells showed the immune infiltration analysis between high- and low-YAP1 expression groups of patients with SKCM. **(C)** Correlations between gene signature model 2 and immune score from TCGA cohort. TCGA, The Cancer Genome Atlas; YAP1, Yes-associated protein 1.

Immune checkpoint inhibitors have been approved for the treatment of melanoma. Since then, they have become one of the most important therapeutic options for patients with melanoma, as they can enhance OS and objective response rates. The differential infiltration of various immune cells in the skin lesions of SKCM is an important factor affecting the effectiveness of immunotherapy. Currently, it is unclear how the expression of YAP1 in SKCM correlates with the differential infiltration of immune cells in the skin lesions. So, we examined the association between YAP1 expression and immune cell infiltration in patients with melanoma. The patients were classified into two subgroups (low- and high-YAP1) according to YAP1 expression. As shown in Figure 6A, we observed distinct infiltration patterns of certain immune cell populations between the high- and low-YAP1 groups. Compared with the low-YAP1 group, the high-YAP1 group exhibited higher infiltration of resting CD4^+^ memory T cells and M2 macrophages, whereas it showed lower infiltration of CD8^+^ T cells, regulatory T (Treg) cells, and activated natural killer cells. On the other hand, other immune cells such as plasma B cells, CD4^+^ naïve T cells, resting natural killer cells, *etc.*, showed no significant differences between the two groups (Figure 6A). Infiltrating immune cells were visualized in the heat map of tumor-infiltrating immune cells for each tumor sample of both subgroups (Figure 6B). Moreover, the correlation between the new prognostic signatures (model 2) and immune score was analyzed with Spearman’s rank correlation coefficient. As shown in Figure 6C, the counts of B cells, CD4^+^ T cells, macrophages, and CD8^+^ T cells were negatively correlated with the risk score.

## 4 Discussion

In the present study, we analyzed data downloaded from the TCGA and GEO databases. The results highlight that Hippo pathway-related genes were differentially expressed between SKCM and normal tissues. We further found a positive correlation between the expression of cuproptosis-related genes and levels of Hippo pathway-related genes in SKCM. Low diagnosis rates and properties for early metastasis are the main reasons responsible for poor prognosis and high mortality. Thus, we decided to conduct a bioinformatics analysis for the identification of effective biomarkers that could assist in early detection of disease. According to the LASSO analyses, a total of 10 Hippo pathway-related genes were identified in this study. Of note, this stepwise dimensionality reduction approach for identifying key prognostic genes and constructing a risk model has been extensively documented in the literature and is a highly reliable method (Guo et al., 2022; Yang et al., 2022). Moreover, subsequent research has confirmed the superior prognostic ability of the risk model. Next, we added cuproptosis-related genes to the prognostic signatures to construct a more effective prognostic signature for melanoma. Surprisingly, the addition of cuproptosis-related genes (i.e., PDHA1, CDKN2A, SLC31A1) into new model improved its clinical usefulness in SKCM.

Targeting the Hippo/YAP/TAZ signaling pathway may provide promising opportunities for cancer therapy. Notably, this pathway acts as a tumor suppressor, and abnormal YAP/TAZ activity has been implicated in various types of skin cancer. In the past, some studies have also revealed that the Hippo pathway may be involved in the mechanisms of melanoma progression. The Hippo pathway is a highly conserved signaling cascade; it was initially identified in *Drosophila melanogaster* as being responsible for larval growth, and later implicated in human cancers as a major tumor suppressor pathway (Ma et al., 2019; Moya and Halder, 2019). The pathway consists of a core kinase cascade beginning with STK4, which phosphorylates and activates large tumor suppressor kinases LATS1 and LATS2 (Hansen et al., 2015; Meng et al., 2016). The Hippo pathway has been identified as a crucial tumor suppressor in melanoma, and YAP/TAZ have emerged as promising therapeutic targets for the treatment of human melanoma in numerous studies. Oncogenic BRAF expression induces activation of the Hippo pathway and cell cycle arrest *in vitro* through a cell-intrinsic mechanism. Hyperactive mitogen-activated protein kinase (MAPK) signaling alters the cytoskeleton, partly by decreasing RhoA signaling, which indirectly leads to Hippo pathway activation (Vittoria et al., 2022). Moreover, studies observed co-heterozygous loss of LATS1/2 and amplification of YAP1 in primary and metastatic human melanoma (Menzel et al., 2014; Zhang et al., 2019).

Copper is implicated in the pathogenesis of cancer, promoting both tumorigenesis and metastasis (Oliveri, 2022). Depending on mitochondrial respiration, copper-induced cell death represents a newly identified form of regulated cell death that is distinct from other well-characterized regulated cell death modalities, including apoptosis, ferroptosis, and necroptosis (Tsvetkov et al., 2022). Under normal circumstances, cells maintain proper levels of intracellular copper through homeostatic mechanisms. These regulatory mechanisms prevent excessive copper accumulation, thereby avoiding cellular damage. An imbalance between intracellular and extracellular copper levels can cause irreversible damage to tumor tissue (Liu et al., 2022). Recently, several *in vitro* experiments have demonstrated that FDX1 promotes the growth and migration of melanoma cells (Liu et al., 2022). This finding suggests that FDX1, which functions as a cuproptosis-related gene, serves as a diagnostic indicator for melanoma. Melanoma is more likely to occur in individuals who express high levels of FDX1; hence, this gene could serve as a prognostic marker for patients with melanoma. At present, some studies suggested that the Hippo pathway might have relationship with ferroptosis in cancer (Ren et al., 2022). Currently, no studies have demonstrated the regulatory role of the Hippo pathway in cuproptosis. Therefore, an innovative aspect of our research is the discovery that the Hippo pathway have a regulatory effect on cuproptosis.

Owing to its potential for early metastasis, melanoma is the most fatal type of skin cancer (Nikolaou and Stratigos, 2014). The interaction between genetic susceptibility and environmental exposure gives rise to multiple risk factors (Rastrelli et al., 2014). The surgical treatment of early melanoma is effective; however, advanced melanoma has a dismal prognosis, rendering surgery an unsuitable option for many patients. For these patients, a treatment plan involving immunotherapy, radiotherapy, chemotherapy, or a combination of these therapies may be considered (Altonsy et al., 2020; Wang et al., 2021). The treatment of malignant melanoma has significantly improved with the advent of targeted therapies and immune checkpoint blockade agents. Nevertheless, the challenge of primary and acquired resistance to treatment persists (Sosman et al., 2012; Postow et al., 2015). Therefore, constructing a prognostic model capable of distinguishing SKCM based on a large sample size dataset has significant clinical value. TCGA and GEO, among other large-scale public databases, provide transcriptome profiles for numerous types of cancer. Use of the GEO database, which contains expression profile data of a wide range of cancers, has enabled scientists to rapidly identify genes closely associated with cancer (Song et al., 2020). In recent years, the application of machine learning to life sciences has become increasingly common. Numerous studies have demonstrated that machine learning plays a crucial role in mining new information from medical big data (Hong et al., 2020; Nayarisseri et al., 2021; Panja et al., 2021). The use of machine learning assists in predicting the occurrence and prognosis of cancer; moreover, this approach can lead to the discovery of new biomarkers of cancer (Chen et al., 2022). Therefore, an extensive body of research has investigated the prognosis and metastasis of melanoma. Thus far, research focused on predicting the prognosis of patients with melanoma based on the expression levels of pyroptotic genes and the tumor microenvironment status (Huang et al., 2020; Wang et al., 2020; Ju et al., 2021). It may be possible to better understand the molecular mechanism underlying the progression of SKCM by identifying the signaling pathways that differ between risk stratification groups. In this study, we also constructed two prognostic models that can effectively differentiate between melanoma patients with favorable and unfavorable prognoses, providing assistance for clinical diagnosis and treatment.

Immunotherapy is a crucial treatment modality for melanoma. The difference in immune cell infiltration is a key factor in determining the effectiveness of immunotherapy. By analyzing the degree of immune cell infiltration in different YAP1 expression subgroups, we identified significant differences in various cells such as Treg cells and M2 macrophages between the two groups. Treg cells, characterized by the expression of fork head box P3 (FOXP3), play a crucial role in maintaining self-tolerance by suppressing immune responses to both self- and non-self antigens in an antigen-nonspecific manner (Fontenot et al., 2005; Overacre-Delgoffe et al., 2017). The lack of Treg cells can result in evident autoimmune disease. However, tumor-infiltrating Treg cells are associated with poor clinical outcomes. It has been shown that they promote cancer progression due to their ability to inhibit antitumor immunity (Facciabene et al., 2012). Therefore, they pose a major obstacle to the success of immunotherapy (Arce Vargas et al., 2017). The depletion of tumor-infiltrating Treg cells exerted synergistic effects with an immune checkpoint inhibitor for tumor eradication (Van Damme et al., 2021). The metabolic pathways of M1 and M2 macrophages exhibit distinct characteristics. Over the past few years, extensive studies have demonstrated that macrophages exhibit a complete tricarboxylic acid cycle, which provides electron transport chain substrate (Viola et al., 2019). Tumor-associated macrophages are exposed to low oxygen and glucose concentrations and AMP-activated protein kinase (AMPK) activation, thereby promoting their anti-inflammatory properties (McGettrick and O'Neill, 2020). A negative correlation was found between YAP1 expression and Treg cell infiltration. However, a positive correlation was found between YAP1 expression and M2 macrophage infiltration. Hence, upregulation of YAP1 might enhance the response to immunotherapy by inhibiting Treg cell infiltration into the tumor microenvironment.

In conclusion, we found that eight Hippo pathway-related genes were downregulated in melanoma, exhibiting prognostic value. Furthermore, YAP1 was positively correlated with M2 macrophages and negatively correlated with Treg cell infiltration. There was a significant positive correlation between cuproptosis-related genes and Hippo pathway-related genes in SKCM. The ability to predict the prognosis of patients with SKCM could be improved following the addition of Hippo pathway-related genes into the novel risk model based on cuproptosis-related genes. Moreover, downregulation of YAP1 could lead to lower expression of FDX1. The presence of this factor may serve as a prognostic indicator for patients with melanoma undergoing immunotherapy.

## Data Availability

The datasets presented in this study can be found in online repositories. The names of the repository/repositories and accession number(s) can be found in the article/Supplementary Material.
