# Supplementary material for: Role of hippo pathway and cuproptosis-related genes in immune infiltration and prognosis of skin cutaneous melanoma
Source: Front Pharmacol. 2024 Mar 7;15:1344755. doi: 10.3389/fphar.2024.1344755 (PMC10955143; doi:10.3389/fphar.2024.1344755)
Supplement: Supplementary file 1 [file Table1.DOCX]

Supplementary Material


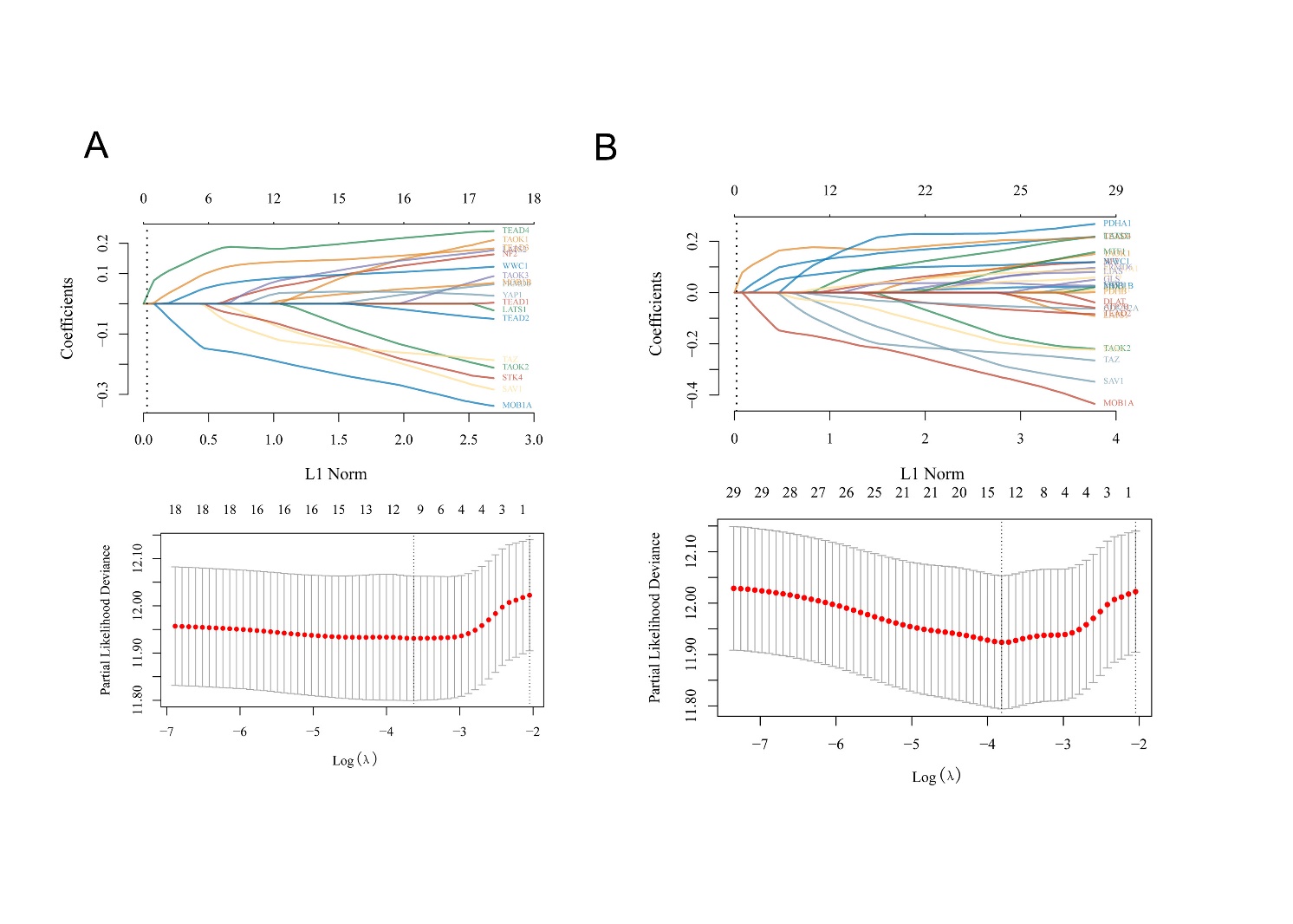


**Supplementary Figure 1.** The coefficients of selected prognostic signatures. The abscissa represents the value of lambda, and the ordinate represents the coefficients of the independent variable.


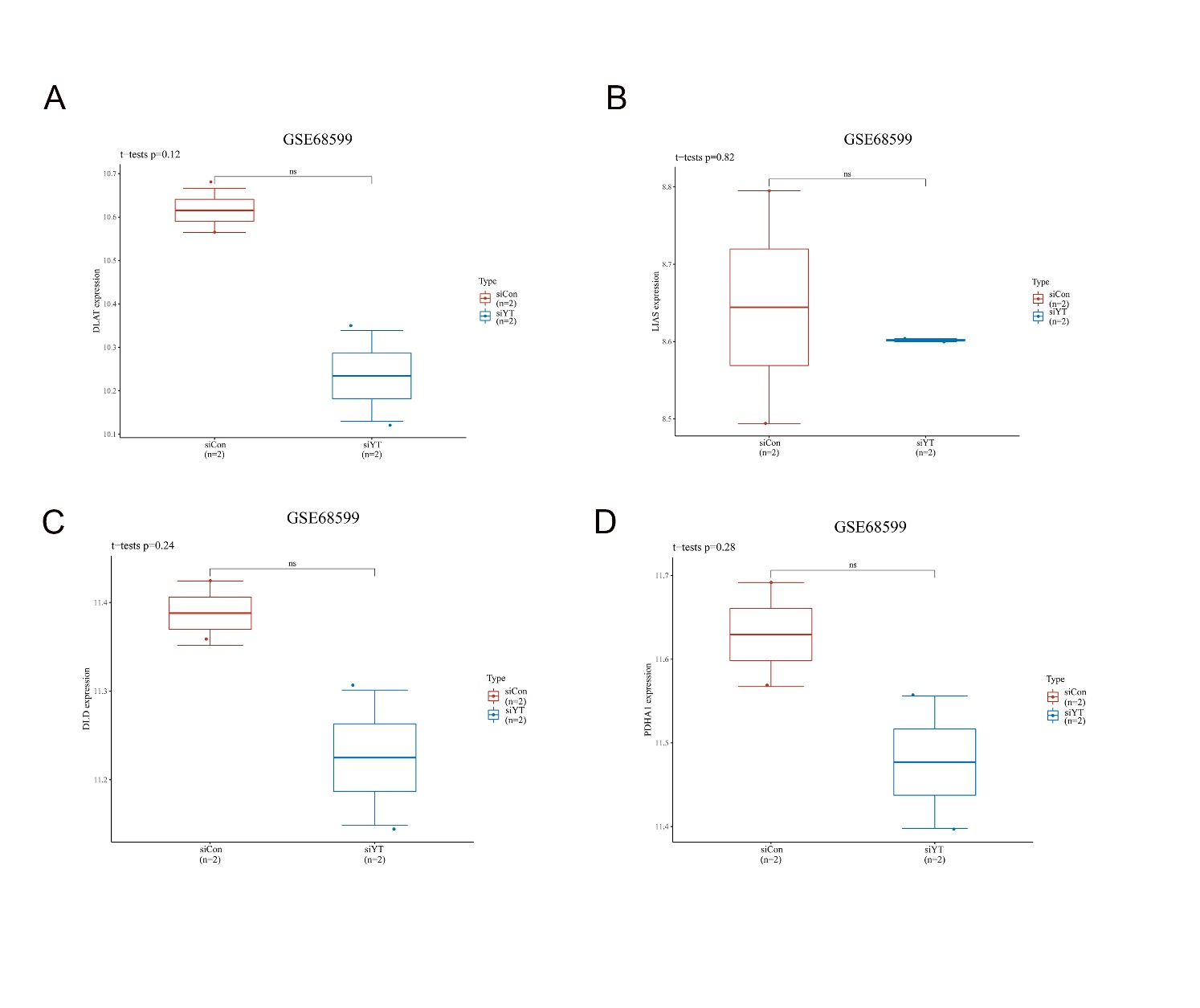


**Supplementary Figure 2.** Analysis of cuproptosis-related genes expression in WM3248 cells. (A-D) The expression of DLAT, LIAS, DLD, PDHA1 after transfection of resistant WM3248 cells with siYAP1 in GSE68599.
